# Metabolic Profiling Based Quantitative Evaluation of Hepatocellular Metabolism in Presence of Adipocyte Derived Extracellular Matrix

**DOI:** 10.1371/journal.pone.0020137

**Published:** 2011-05-16

**Authors:** Nripen S. Sharma, Deepak Nagrath, Martin L. Yarmush

**Affiliations:** 1 Center for Engineering in Medicine/Surgical Services, Massachusetts General Hospital, Harvard Medical School, and The Shriners Hospitals for Children, Boston, Massachusetts, United States of America; 2 Department of Chemical and Biomolecular Engineering, Rice University, Houston, Texas, United States of America; Rutgers University, United States of America

## Abstract

The elucidation of the effect of extracellular matrices on hepatocellular metabolism is critical to understand the mechanism of functional upregulation. We have developed a system using natural extracellular matrices [Adipogel] for enhanced albumin synthesis of rat hepatocyte cultures for a period of 10 days as compared to collagen sandwich cultures. Primary rat hepatocytes isolated from livers of female Lewis rats recover within 4 days of culture from isolation induced injury while function is stabilized at 7 days post-isolation. Thus, the culture period can be classified into three distinct stages viz. recovery stage [day 0–4], pre-stable stage [day 5–7] and the stable stage [day 8–10]. A Metabolic Flux Analysis of primary rat hepatocytes cultured in Adipogel was performed to identify the key metabolic pathways modulated as compared to collagen sandwich cultures. In the recovery stage [day 4], the collagen-soluble Adipogel cultures shows an increase in TriCarboxylic Acid [TCA] cycle fluxes; in the pre-stable stage [day 7], there is an increase in PPP and TCA cycle fluxes while in the stable stage [day 10], there is a significant increase in TCA cycle, urea cycle fluxes and amino acid uptake rates concomitant with increased albumin synthesis rate as compared to collagen sandwich cultures throughout the culture period. Metabolic analysis of the collagen-soluble Adipogel condition reveals significantly higher transamination reaction fluxes, amino acid uptake and albumin synthesis rates for the stable vs. recovery stages of culture. The identification of metabolic pathways modulated for hepatocyte cultures in presence of Adipogel will be a useful step to develop an optimization algorithm to further improve hepatocyte function for Bioartificial Liver Devices. The development of this framework for upregulating hepatocyte function in Bioartificial Liver Devices will facilitate the utilization of an integrated experimental and computational approach for broader applications of Adipogel in tissue e engineering and regenerative medicine.

## Introduction

Hepatocytes constitute about 70% of the cellular population of the liver and play an indispensable role in over 500 metabolic, regulatory and immune functions [1] including plasma protein synthesis, bile production, nutrient regulation and xenobiotic detoxification [2]. In cases of irreversible liver failure, such as cirrhosis and fulminant hepatic failure, a promising *in vitro* system to maintain hepatic function is vital. Extracorporeal bioartificial liver devices (BAL) are perhaps among the most promising technologies for the treatment of liver failure, but significant technical challenges remain in order to develop *in vitro* systems with sufficient functional capacity [3]. Such *in vitro* systems are also imperative for drug metabolism and toxicity evaluation studies [4].

Various methodologies to maintain hepatocytes *in vitro* include effect of extracellular matrix topology, cellular environment and medium composition on function [5]–[22]. The traditional technique for culturing rat hepatocytes *in vitro* is the collagen I sandwich configuration [23]–[26] While this system has been extensively characterized with expression of basolateral and apical markers, upregulation of differentiated function and maintenance of cell polarity; there is also evidence for the role of extracellular matrix composition on maintenance of cell function [27].

*In vivo*, the hepatic ECM also known as the Space of Disse is a 1–1.5 mm complex macromolecular deposition of collageneous proteins, fibronectin, entactin and heparin sulfate proteoglycans [28] between the membranes of the endothelial cells and the hepatocytes thus comprising the hepatic interstitium. The hepatic ECM has gained considerable interest in modulation of hepatic differentiated function, morphological polarization, liver regeneration, hepatogenesis and the progression of liver cirrhosis. Hepatic ECM co-ordinates cytokine secretion and clearance as well as initiating cell surface receptor–matrix polypeptide interactions. These physiological components have a dramatic effect on cellular function and metabolism.

Thus, influence of ECM on hepatocyte culture systems is important from two perspectives. From an application standpoint, development of scalable, simple, “in vivo” like and cost-effective *in vitro* systems for hepatic functional upregulation in Bioartificial Liver Devices and drug toxicity studies is necessary. From a mechanistic standpoint, these systems are useful to unravel the effect of ECMs on hepatic function and metabolism.

Variation in ECM compositions including addition of glycosaminoglycans and hepatic proteoglycans that promotes formation of gap junctions [29]. EHS biomatrix Matrigel cultures has also been used as a substitute for collagen I that maintains hepatocyte polarity [30] and induces expression of cell adhesion molecules viz. connexins with upregulation of differentiated function comparable to double gel cultures [31]. Matrigel, prepared from extract of murine EHS tumors is comprised primarily of collagen IV, laminin, perlecan, nidogen, FGF, EGF and IGF [32], [33]. While EHS tumor derived matrix components viz. collagen IV and heparan sulfate proteoglycan are prevalent in the Space of Disse, utilizing a matrix that resembles the hepatic ECM [34], [35] will induce improved differentiated function similar to the *in vivo* microenvironment [28], [36], [37].

Moreover, the developed *in vitro* system will provide a platform to assess the effect of hepatic-like ECM on cell metabolism and function. The inter-relationship between hepatic intracellular pathways and the ECM is currently less studied and the elucidation of the effect of the ECMs on the metabolism is critical to identify the mechanism of *in vivo* hepatocellular functional and morphological integrity. This necessitates a suitable *in vitro* model system that can closely mimic the *in vivo* hepatic microenvironment.

We have previously developed a system to synthesize mammalian preadipocyte cell secreted extracellular matrix proteins termed Adipogel in copius amounts. Adipogel has been shown to exhibit at least fibronectin [the most abundant ECM in the Space of Disse] and collagen IV that is present in the Space of Disse. We have also shown that Adipogel laden on top of collagen single gel hepatocyte cultures increased albumin differentiated function as compared to collagen double gel cultures [38]. We hypothesized that increased albumin synthesis in hepatocyte cultures as compared to collagen double gel cultures may be due to increased levels of laminin, fibronectin and Collagen IV in Adipogel as compared to absence of these factors in double-gel sandwich configurations. Literature evidence show the effects of these components in augmenting albumin synthesis rates [23], [39], however, the combined effects of these components has not been thoroughly investigated. Thus, the collagen-Adipogel system of hepatocyte culture is a suitable *in vitro* model for investigating the interactions between hepatic ECM and metabolism.

Numerous researchers have investigated the intermediary metabolism of hepatocytes, the primary functional cells of the liver [40]–[42]. Since hepatic metabolism is directly linked to cellular energetic and functions, the effect of hepatocyte culture configurations on the biochemical pathways inherent in mature cells is critical. While there has been substantial work on the characterization of intermediary metabolism of hepatocytes in the presence of growth factor or metabolite supplementations [43]–[49]. little knowledge is available on the effect of extracellular matrices on hepatic metabolism [27]. The elucidation of the pathway fluxes differentially upregulated or downregulated in the presence of ECM based hepatocyte cultures will provide a methodology to identify the effect of matrix components on hepatocellular metabolism for augmented function. In the current work, we have developed a framework for Metabolic Flux Analysis of hepatocyte cultures in the presence of Adipogel at various time points of the differentiation process. This framework provides a characterization of the intracellular metabolite flux changes in response to a natural extracellular matrix supplementation.

## Materials and Methods

### Materials

Dulbecco's Modified Eagle Medium [DMEM] containing 4.5 g/L glucose, fetal bovine serum [FBS], penicillin, streptomycin, and trypsin–EDTA were obtained from Invitrogen Life technologies [Carlsbad, CA]. Dexamethasone, isobutyl-methylxanthine, epidermal growth factor, insulin, glucagon, rosiglitazone and hydrocortisone were purchased from Sigma-Aldrich [St. Louis, MO]. 100 kDa cutoff protein centrifugal filters were purchased from Millipore Technologies [Billerica, MA].

### Adipogel Generation using Preadipocyte Differentiation

Adipogel was generated from differentiating murine preadipocytes as described previously. Briefly, 3T3-L1 murine preadipocytes were cultured in T-175 cm^2^ flasks in Dulbecco's Modified Eagle's media supplemented with 10% FBS, 2% Penicillin and Streptomycin till the cells attained confluency. 48 hours post confluency, the cells were differentiated in culture media supplemented with 1 µM dexamethasone, 0.1 µM isobutyl-methylxanthine and 1 µM rosiglitazone for 2 days with media changes every two days. On the second day post-differentiation, cells were exposed to culture medium supplemented with 1 µM rosiglitazone for an additional 2 days. Media supernatant was collected on days 2 and 4 of differentiation and stored at 4°C prior to further processing.

**Figure 1:**
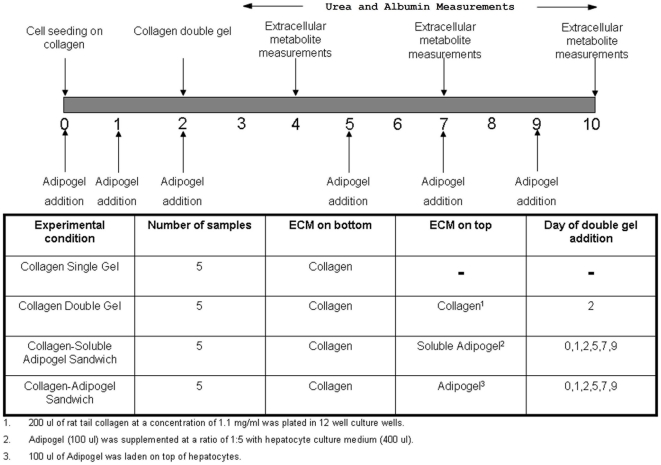
Experimental design of hepatocyte cultures. Four different conditions were utilized for the metabolite measurements. Collagen single gel [CSG], collagen double gel [CDG] collagen-soluble Adipogel sandwich [CSG+solASG] and collagen-Adipogel sandwich cultures [CSGASG]. Secreted products were measured at the recovery stage, pre-stable stage and stable stage of culture. Urea and albumin synthesis was determined from day 3 to day 10 of culture.

In order to purify the extracellular matrix rich material, the differentiated preadipocyte conditioned media was centrifuged at 4000*g* for 1.5 hr using an Amicon 100 kDa centrifugal filter. Since the concentrate was derived from adipocyte-related cell type and had a gel-like configuration, it was termed as ‘Adipogel’. To elucidate the effect of Adipogel on hepatocellular metabolism, four different conditions were tested as described in Figure 1. The four conditions were [i] collagen single gel cultures, [ii] collagen double gel cultures, [iii] collagen single gel with soluble Adipogel on top of hepatocytes and collagen single gel with Adipogel on top of hepatocytes [Figure 1]. To obtain a metabolic profile of hepatocytes under these conditions, 3 different time points were chosen viz. the recovery stage [day 0–4], the pre-stabilization stage [day 5–7] and the stabilization stage [day 8–10].

### Primary Rat Hepatocyte Isolation

Female Lewis rats [Charles River Laboratories, Wilmington, MA] weighing 180 to 200 g [2 to 3 months old] were used as a hepatocyte source and were maintained in accordance with National Research Council guidelines. Experimental protocols were approved by the Subcommittee on Animal Care, Committee on Research, Massachusetts General Hospital (Protocol # 2005N000130). Using a modification of the two-step collagenase perfusion method [24], which involves purification of the cell suspension by means of centrifugation over Percoll, approximately 100–200 million cells were routinely isolated from one rat with viability between 85 and 98%, as judged by trypan blue exclusion.

### Hepatocyte Culture in Collagen Sandwich and Adipogel

Type 1 collagen was prepared by extracting acid-soluble collagen from Lewis rat-tail tendons. To create a thin layer of collagen gel in 12-well tissue culture plates, 400 µl of an ice-cold mixture of 1 part of 10× concentrated DMEM and 9 parts of 1.25 mg/ml rat tail tendon type I collagen were evenly distributed over the bottom of each well. The plates were incubated at 37°C for 60 min to induce collagen gelation before cell seeding. Each well of the 12-well culture plates received 5×10^5^ primary hepatocytes in suspension in 0.5 ml standard hepatocyte culture medium, which consisted of DMEM supplemented with 14 ng/ml glucagon, 7.5 µg/ml hydrocortisone, 0.5 U/ml insulin, 20 ng/ml EGF, 200 U/ml penicillin, 200 µg/ml streptomycin, and 10% FBS. Cultures were incubated in 90% air/10% CO_2_ at 37°C. Cells were rinsed with 1× PBS to remove non-adherent cells 4–6 hours after seeding. For the double collagen gel culture configuration, a second layer of 250 µl collagen was laden on top of the cells 48 hours post-seeding [Figure 1]. Medium was changed every 24 hours and collected from day 3 onwards until day 10. Three additional culture conditions were utilized as described below. For the Adipogel conditions, the basement membrane extract [BME] isolated on Day 4 was utilized for the entire set of hepatocyte experiments. For the soluble Adipogel condition, 100 µl of Adipogel was solubilized in 400 µl of culture medium by continuous pipeting. The supplemented media was added to cell cultures on days 0, 1, 2, 5, 7 and 9 of cultures. For the second condition, to form the adipocyte derived gel, 100 µl of Adipogel was uniformly spread over each well by slow dripping along the wall. To promote gelation, the plates were incubated at 37°C for 60 min followed by addition of culture medium.

### Hepatocyte Functional Assessment

Albumin concentration in the collected medium samples was analyzed using a competitive enzyme-linked immunosorbent assay [ELISA]. Albumin protein and the antibody were purchased from MP Biomedicals. Urea concentration was determined via its specific reaction with diacetyl monoxime with a commercially available assay kit [Fisher Scientific, Pittsburgh, PA]. The absorbance was measured with a Thermomax microplate reader [Molecular Devices, Sunnyvale, CA]

### Biochemical Assays

The biochemical assays were performed at the recovery, pre-stable and stable stages of culture with media samples. Amino acids were fluorescently labeled using the AccQ-Tag system [Waters Co., Milford, MA], separated by high performance liquid chromatography [HPLC Model 2690, Waters Co.], and quantitated by a fluorescence detector [Model 474, Waters Co.]. Glucose and lactate levels were measured with commercially available kits [Sigma], the former based on the reaction of glucose catalyzed by glucose oxidase and the latter based on the conversion of lactate to pyruvate catalyzed by lactate oxidase. Acetoacetate and β-hydroxybutyrate were measured using a commercially available kit [Bioassay Systems].

### Metabolic Flux Analysis

MFA is a useful methodology to characterize the differential activation of metabolic pathways in hepatocyte cultures. Thus, based on a stoichiometric model for the metabolic reaction network prevalent in hepatocytes, intracellular reaction fluxes are estimated by mass balances around each intracellular metabolite and extracellular flux measurements. This gives the possibility to calculate intracellular metabolite fluxes, which are difficult to measure from relatively few measurements and to corroborate the metabolic network. The model used in this work was originally developed for perfused liver [50] and modified subsequently for cultured hepatocytes with incorporation of lipid metabolism reactions [45]. Figure 3 illustrates the metabolic network considered. Table S1 and Table S2 show the list of reactions and metabolites included. The main assumptions for the application of MFA to the hepatic metabolic network are as follows [45], [51]:

1. The metabolic network is based on known stoichiometry of hepatic intermediary metabolism with consideration of carbon and nitrogen balances.
2. Albumin is a major protein product of hepatocytes and hence only this protein is considered.
3. The cellular uptake/secretion rates of metabolites are distinct from the intracellular fluxes of the corresponding metabolites. Thus, the intracellular and extracellular pools of substrates have been distinguished. Also, the mechanisms of active and passive transport have not been incorporated.
4. The metabolite pools are at pseudo steady-state with a single pool in the cell. The influx and efflux of metabolites into/from hepatocytes are calculated from the amount of metabolites remaining in the extracellular media after 24 h.

Following the above assumptions, the mathematical model consists of mass balances around 45 intracellular metabolites [Table 1] considering 72 reactions [Table S1, Table S2 and Figure 3]. The sum of fluxes to and from the metabolite pools is assumed to be zero [pseudo steady-state assumption]:
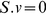
(1)where the matrix S contains the stoichiometric coefficients of the incorporated reactions. Each element S_ij_ of S is the coefficient of metabolite i in reaction j, and each v_j_ of vector v is the net flux or conversion rate of reaction j. Equation 1 is separated into measured and unknown fluxes, v_m_ and v_u_, respectively, as follows:
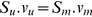
(2)The measured fluxes represent measured rates of uptake or release of extracellular metabolites and thus, solving Equation 2 gives estimates of intracellular fluxes.

**Table 1:** List of intracellular metabolites for mass balance.

MetabolitePools
Glucose-6Phosphate
Ribulose-5Phosphate
Ribose-5Phosphate
Xylulose-5Phosphate
Erythrose-4Phosphate
Glyceraldehyde-3Phosphate
Fructose-6Phosphate
Fructose-1,6BisPhosphate
PhosphoEnolPyruvate
Pyruvate
NADH
FADH2
Acetyl-CoA
Oxaloacetate
Citrate
2-oxo-gluterate
Succinyl-CoA
Fumarate
Malate
Ammonia
Ornithine
Citrulline
Acetoacetyl-CoA
Acetoacetate
Alanine
Cysteine
Aspartate
Glutamate
Phenylalanine
Glycine
Histidine
Isoleucine
Lysine
Leucine
Methionine
Asparagine
Proline
Glutamine
Arginine
Serine
Threonine
Valine
Tyrosine
CO2
Oxygen

A total of 45 metabolites have been listed in the table.

### Statistical analysis

Each data point represents the mean of two or three experiments [each with three biological replicates], and the error bars represent the standard error of the mean. Data was normalized to number of cells initially seeded and expressed as µmol/million cells/day.

For extracellular metabolite measurements, the data-sets for each metabolite flux for each experimental condition were averaged from the sum of all replicates per experimental condition. The standard error of the mean was calculated from replicate data-set for each experimental condition. The mean and standard error of the mean of the extracellular metabolite measurements is quantitatively represented in the Table 2, Table 3 and Table 4 and qualitatively represented in Figure 4.

**Table 2:** Effect of Adipogel substrate on amino acid and glucose metabolism of recovery stage hepatocyte cultures.

RecoveryStage					
Fluxno.	Metabolites	CSG	CDG	CSG+solASG	CSG+ASG
1	Glucose	−0.41±0.016	−0.388±0.362	−0.873±0.495	−0.373±0.419
14	Lactate	0.582±0.092*	0.743±0.061	1.274±0.555	0.971±0.759
23	Urea	1.952±0.051*	1.479±0.123	1.828±0.442	1.533±0.312
45	βhydroxybutyrate	−0.003±0.016	−0.012±0.002	0.041±0.011	0.019±0.003
48	Albumin	0.0001±0*	0.0002±0	0.0008±0.0004*	0.0003±0.0002
49	O_2_	0.916±0.128	1.169±0.163	2.005±0.28	1.528±0.213
50	CO_2_	0.102±0.018	0.13±0.023	0.223±0.039	0.17±0.029
51	Acetoacetate	0.124±0.005	0.257±0.149	0.094±0.059	0.133±0.097
52	Ornithine	−0.04±0.014*	−0.067±0.015	−0.031±0.027*	−0.04±0.018*
53	Ammonia	−0.562±0.045	−0.599±0.015	−0.646±0.028*	−0.601±0.006
55	Cysteine	0.016±0.067	−0.04±0.02	0.059±0.056*	0.053±0.052*
56	Aspartate	0.017±0.021	0.005±0.005	0.006±0.012	−0.002±0.01
57	Glutamate	0.027±0.075	0.02±0.014	0.042±0.027	0.032±0.011
58	Phenylalanine	−0.049±0.035	−0.052±0.015	−0.126±0.041*	−0.109±0.015*
59	Glycine	−0.163±0.075	−0.167±0.01	−0.209±0.026*	−0.206±0.017*
60	Histidine	−0.256±0.121	−0.176±0.04	−0.511±0.179*	−0.298±0.038*
61	Isoleucine	−0.025±0.025	−0.03±0.016	−0.092±0.042*	−0.073±0.023*
62	Lysine	−0.116±0.083	−0.159±0.059	−0.285±0.097*	−0.244±0.051*
63	Leucine	−0.03±0.031	−0.036±0.019	−0.135±0.087*	−0.085±0.025*
65	Asparagine	−0.008±0	−0.005±0	0±0	−0.004±0
66	Proline	−0.03±0.02	−0.027±0.003	−0.012±0.027	−0.002±0.021*
67	Glutamine	8.017±22.261	0.887±0.627	0.183±0.118	0.87±0.287
68	Arginine	−0.021±0.071	0.139±0.227	0.183±0.118*	−0.006±0.03
69	Serine	−0.104±0.066	−0.08±0.018	−0.052±0.052*	−0.13±0.004*
70	Threonine	−0.277±0.105	−0.316±0.035	−0.165±0.046*	−0.375±0.023*
71	Valine	−0.018±0.031	−0.025±0.027	−0.373±0.018*	−0.078±0.027*
72	Tyrosine	−0.169±0.007*	−0.146±0.01	−0.135±0.085*	−0.185±0.009*

Hepatocytes were cultured in four different configurations at a density of 500,000 cells/well in a 12 well plates for 10 days. *Values are in µmol/million cells/day.*

*indicates p<0.05 for each experimental condition vs. CDG. Sign conventions are in accordance with Table S1. −ve value for glucose corresponds to glucose uptake; −ve value for amino acids corresponds to amino acid uptake; +ve value for acetoacetate corresponds to acetoacetate release.

**Table 3:** Effect of Adipogel substrate on amino acid and glucose metabolism of pre-stable stage hepatocyte cultures.

Pre-stableStage					
Fluxno.	Metabolites	CSG	CDG	CSG+solASG	CSG+ASG
1	Glucose	0.37±0.046	0.222±0.274	−0.03±0.047*	0.297±2.35
14	Lactate	0.393±0.359	0.001±0	0.883±0.196	0.637±0.546*
23	Urea	0.97±0.06*	1.852±0.13	1.869±0.135	1.555±0.486
45	βhydroxybutyrate	−0.021±0.01	−0.012±0.016	0.026±0.003	−0.01±0.003
48	Albumin	0.0001±0*	0±0	0.001±0.001*	0.001±0*
49	O_2_	0.619±0.086	0.002±0.001	1.39±0.194	1.003±0.14
50	CO_2_	0.062±0.009	0.0002±0.0001	0.139±0.02	0.1±0.014
51	Acetoacetate	−0.054±0.014	−0.011±0.025	0.054±0.013*	−0.051±0.023
52	Ornithine	−0.048±0.016	−0.082±0.003*	−0.023±0.013	−0.066±0.01*
53	Ammonia	−0.394±0.135	−0.638±0.008*	−0.645±0.047	−0.615±0.03
55	Cysteine	−0.007±0.059	−0.042±0.005	0.083±0.02*	0.008±0.024*
56	Aspartate	0.002±0.011	0.004±0.006	0±0.009	0.006±0.016
57	Glutamate	0.021±0.049	0.025±0.008	0.031±0.043	0.044±0.034
58	Phenylalanine	−0.058±0.049	−0.143±0.015*	−0.164±0.012*	−0.127±0.037
59	Glycine	−0.192±0.03	−0.22±0.018	−0.205±0.035	−0.179±0.046
60	Histidine	−0.374±0.172	−0.468±0.03	−0.537±0.081	−0.44±0.098
61	Isoleucine	−0.07±0.058	−0.13±0.116	−0.118±0.064	−0.073±0.053
62	Lysine	−0.156±0.16	−0.285±0.019	−0.312±0.058	−0.25±0.07
63	Leucine	−0.074±0.065	−0.087±0.018	−0.138±0.065	−0.086±0.058
65	Asparagine	−0.007±0.002	−0.002±0.003*	−0.001±0.002	−0.004±0*
66	Proline	−0.002±0.01	−0.005±0.003	−0.025±0.022	−0.016±0.017
67	Glutamine	6.395±14.477	1.121±0.377	0.136±0.188*	1.198±0.917
68	Arginine	−0.021±0.039	−0.007±0.021	−0.062±0.052	0.017±0.078
69	Serine	−0.073±0.101	−0.152±0.01	−0.181±0.026	−0.142±0.032
70	Threonine	−0.269±0.228	−0.378±0.008	−0.378±0.011	−0.363±0.016
71	Valine	−0.072±0.073	−0.081±0.02	−0.129±0.074	−0.07±0.073
72	Tyrosine	−0.095±0.068	−0.122±0.024	−0.166±0.041	−0.107±0.068

Hepatocytes were cultured in four different configurations at a density of 500,000 cells/well in a 12 well plates for 10 days. *Values are in µmol/million cells/day.*

*indicates p<0.05 for each experimental condition vs. CDG. Sign conventions are in accordance with Table S1. −ve value for glucose corresponds to glucose uptake; −ve value for amino acids corresponds to amino acid uptake; +ve value for acetoacetate corresponds to acetoacetate release.

**Table 4:** Effect of Adipogel substrate on amino acid and glucose metabolism of stable stage hepatocyte cultures.

StableStage					
Fluxno.	Metabolites	CSG	CDG	CSG+solASG	CSG+ASG
1	Glucose	0.072±0.016*	−0.001±0.04	0.073±0.043*	0.072±0.0358*
14	Lactate	0.071±0.022	0.068±0.015	0.095±0.013*	0.103±0.039
23	Urea	1.074±0.77*	1.946±0.155	1.847±0.253	1.703±0.299
45	βhydroxybutyrate	−0.021±0.005	−0.021±0.005	0.073±0.022	0.073±0.022
48	Albumin	0±0*	0.0007±0.0001	0.0017±0.0002*	0.0008±0.0002
49	O_2_	0.112±0.016	0.107±0.015	0.149±0.021	0.162±0.023
50	CO_2_	0.013±0.003	0.012±0.002	0.017±0.003	0.018±0.003
51	Acetoacetate	0.071±0.009	0.071±0.009	−0.024±0.014	−0.024±0.014
52	Ornithine	−0.052±0.016*	−0.082±0.003	−0.025±0.01*	−0.066±0.011*
53	Ammonia	0.346±0.545	−0.005±0.006	−0.045±0.007	0.005±0.011
55	Cysteine	0.017±0.052	0.042±0.005	−0.088±0.018	−0.008±0.027*
56	Aspartate	0.005±0.002	0.003±0.001	0.001±0.001	0.003±0.001
57	Glutamate	0.048±0.022*	0.142±0.006	0.085±0.01	0.092±0.021*
58	Phenylalanine	−0.001±0.072*	−0.198±0.011	−0.252±0.01	−0.167±0.012*
59	Glycine	0.032±0.083	−0.035±0.014	−0.092±0.015*	−0.006±0.037
60	Histidine	−0.034±0.019*	−0.091±0.001	−0.092±0.001	−0.086±0*
61	Isoleucine	0.037±0.109*	0.172±0.017	−0.133±0.036*	−0.019±0.057*
62	Lysine	0.038±0.073	−0.003±0.015	−0.096±0.013*	−0.041±0.017*
63	Leucine	0.021±0.098*	0.134±0.017	−0.164±0.032*	−0.045±0.051*
65	Asparagine	0.006±0.003*	0±0	−0.003±0.002*	0.004±0*
66	Proline	0.325±0.343	0.021±0.006	0.069±0.014*	0.063±0.006*
67	Glutamine	13.714±3.82*	6.444±0.746	0.125±0.518*	2.496±1.908*
68	Arginine	0.001±0.002*	0.008±0.001	−0.003±0.002*	−0.001±0.002*
69	Serine	0.027±0.035*	−0.038±0.004	−0.061±0.005*	−0.03±0.003*
70	Threonine	−0.046±0.047*	−0.188±0.002	−0.411±0.617	−0.73±0.94*
71	Valine	0.051±0.108*	0.203±0.014	−0.222±0.35*	0.007±0.057*
72	Tyrosine	0.002±0.072*	0.104±0.02	−0.116±0.026*	0.03±0.047*

Hepatocytes were cultured in four different configurations at a density of 500,000 cells/well in a 12 well plates for 10 days. *Values are in µmol/million cells/day.*

*t12indicates p<0.05 for each experimental condition vs. CDG. Sign conventions are in accordance with Table S1. −ve value for glucose corresponds to glucose uptake; −ve value for amino acids corresponds to amino acid uptake; +ve value for acetoacetate corresponds to acetoacetate release.

The MFA framework was applied to each biological experimental data-set of extracellular metabolite measurements in µmol/million cells/day input units. The output of the MFA for each biological experimental data-set was averaged using the sum over all computationally derived replicates for the unknown [non-measured] fluxes. The standard error of the mean was calculated from the computationally derived replicates for the unknown [non-measured] fluxes.

The summary statistics were calculated using t-test by comparing data from all experiments from one experimental condition for (e.g.: CSG+solASG) vs. data from all experiments from another experimental condition (for e.g.: CDG). This statistical method was used for extracellular metabolite measurements and computed intracellular fluxes while comparing a specific metabolite flux between different experimental conditions. Statistical significance was determined using the Student's t-test for unpaired data. Differences were considered significant when the probability was less than or equal to 0.05.

## Results

### Development of system to generate Adipogel

We have previously performed a preliminary characterization of the extracellular matrix components derived as a basement membrane extract from preadipocytes during the differentiation process [38].

The preadipocytes are cultured in T-175 cm^2^ flasks in Dulbecco's Modified Eagle's media supplemented with 10% FBS, 2% Penicillin and Streptomycin till the cells attain confluency. 48 hours post confluency, the cells are differentiated in culture media supplemented with 1 µM dexamethasone, 0.1 µM isobutyl-methylxanthine and 1 µM rosiglitazone for 2 days with media changes every two days. On day 2, the differentiation medium is supplemented with rosiglitazone only.

During the differentiation process, cell exposed media is collected and processed further for generation of cell derived extracellular matrix. We have previously identified a highly viscoelastic material on days 2 and 4 of adipocyte differentiation resembling extracellular matrix components secreted by preadipocytes to maintain adipose tissue cell-cell contact, morphological induction of adipocytes and functional and gene expression indicative of mature adipocyte lineage. In order to purify the extracellular matrix-rich material, the cell exposed media is centrifuged at 4000*g* for 1.5 hr using an Amicon 100 kDa or 10 kDa centrifugal filters. The concentrate, primarily composed of media constituents with molecular cut-off of 100 kDa or 10 kDa comprises the cell culture supernatant derived protein rich extracellular matrix.

### Effect of Adipogel on Hepatic Differentiated Function

**Figure 2:**
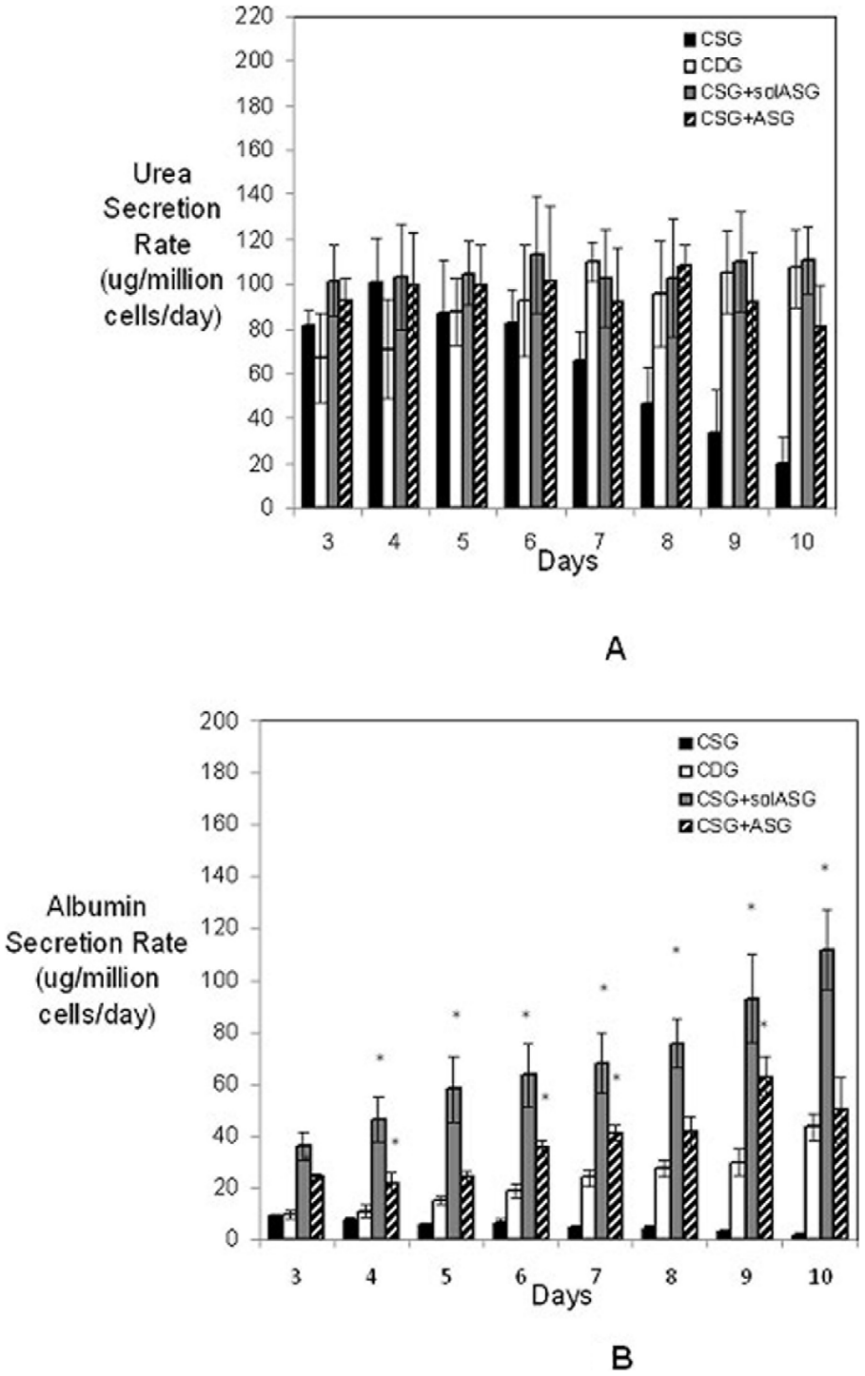
Functional Analysis of Rat Hepatocytes *In vitro* Using Adipogel. [A] Urea and [B] Albumin Secretion rate of Hepatocytes cultured in five different configurations at a density of 500,000 cells/well in a 12 well plate; CSG corresponds to culture on single collagen gel; CDG corresponds to culture in collagen double gel sandwich configuration; CSG+solASG corresponds to hepatocytes cultured on collagen single gel with soluble Adipogel in the media; CSG+ASG corresponds to culture on collagen single gel with Adipogel overlaid on top. Adipogel was utilized at a 1∶5 ratio with culture media and media was changed on days 0, 1,2,5,7 and 9. While the urea secretion rates are similar for the CDG [positive control] and the CSG+solASG conditions, the albumin secretion rate is significantly higher for the CSG+solASG condition as compared to CDG cultures. * indicates p<0.05 vs. CDG condition.

Routine culture of primary hepatocytes is difficult and cumbersome due to their ability to develop compromised function. We have developed a primary hepatocyte culture system that supersedes the traditional methodology of maintaining hepatocyte function and polarity in collagen double gel sandwich systems. Hepatocytes when cultured on single collagen gel with a soluble matrix of Adipogel in the culture media showed comparable urea secretion rates [Figure 2A] but significantly higher albumin secretion rates [Figure 2B] from recovery stage until stable stage of culture as compared to collagen double gel cultures.

### Effect of Adipogel on Hepatic Metabolism

We have performed a metabolic analysis of the different culture configurations viz. the collagen single gel [CSG], collagen double gel [CDG], collagen-Adipogel sandwich [CSG+ASG] and collagen single gel+soluble Adipogel [CSG+solASG]. To elucidate the effect of Adipogel on hepatocyte metabolism, we have compared the CDG to the CSG+solASG conditions as described below. We have chosen day 4 [recovery stage], day 7 [pre-stable stage] and day 10 [stable stage] of analysis to investigate the delayed effect [day4, day7] as well as the immediate effect [day10] of Adipogel supplementation on hepatic metabolism. Primary rat hepatocytes isolated from livers of female Lewis rats recover within 4 days of culture from isolation induced injury while function is stabilized at 7 days post-isolation [52]. Thus, these stages were defined based on stabilization of function for the culture conditions from day 4[recovery] to day 7[pre-stable] to day 10[stable] stage of cultures.

### Glucose and lactate measurements

As shown in Table 3 and Table 5, for the CDG condition, hepatocytes are glycolytic [v_1_] at the recovery stage and stable stage of culture respectively with glucose production at the pre-stable stage [Table 4]. On the other hand, CSG+solASG condition reveals significantly higher glucose consumption at the pre-stable stage and glucose production at the stable stage as compared to CDG condition. The lactate consumption rate [v_14_] is significantly higher for the CSG+solASG condition as compared to the CDG condition only at the stable stage.

**Table 5:** Summary of results for the CSG+solASG condition on different experimental days.

Pathways	FluxNo.	RecoveryStage	Pre-stableStage	StableStage
		CSG+solASG	CSG+solASG	CSG+solASG
Glucose Uptake	1	Base	Decrease significantly	Increase significantly
PPP	2,3,5	Base	Decrease significantly	Same
PPP	4,6	Base	Decrease significantly	Decrease significantly
Gluconeogenesis	7,8	Base	Increase significantly	Same
Gluconeogenesis	9	Base	Same	Increase significantly
Glycerol Uptake	10	Base	Decrease significantly	Decrease significantly
Gluconeogenesis	11–14	Base	Same	Decrease significantly
TCA	15–18	Base	Decrease significantly	Same
Pyruvate Synthesis	24,26	Base	Same	Increase significantly
Amino Acid Metabolism	25,27,28	Base	Same	Decrease significantly
Amino Acid Metabolism	30,31,33,34	Base	Same	Decrease significantly
Amino Acid Metabolism	36,37	Base	Same	Increase significantly
Amino Acid Metabolism	38,40	Base	Same	Decrease significantly
Lipid Metabolism	42–44	Base	Same	Increase significantly
Amino acid Uptake	53–55	Base	Same	Increase significantly
Amino acid Uptake	56	Base	Same	Decrease significantly
Amino acid Uptake	57	Base	Same	Decrease significantly
Amino acid Uptake	58	Base	Decrease significantly	Increase significantly
Amino acid Uptake	59–64	Base	Same	Increase significantly
Amino acid Uptake	65	Base	Same	Decrease significantly
Amino acid Uptake	66	Base	Same	Decrease significantly
Amino acid Uptake	68–72	Base	Same	Increase significantly

Hepatocytes were cultured on collagen single gel at a density of 500,000 cells/well in a 12 well plate. Adipogel was utilized at a 1∶5 ratio with culture media and media was changed on days 0, 1,2,5,7 and 9 of culture. Metabolic Flux Analysis was performed on [A] recovery stage, [B] pre-stable stage and [C] stable stage of culture.

### Amino acid fluxes

At the recovery stage and pre-stable stage, cysteine synthesis [v_55_] is significantly higher for CSG+solASG vs. CDG condition. At the recovery stage, glutamine [v_67_] and arginine synthesis rate [v_68_] is also significantly higher for CSG+solASG vs. CDG condition. At the stable stage, serine [v_69_], glycine [v_59_], isoleucine [v_61_], lysine [v_62_], leucine [v_63_], methionine [v_64_], arginine [v_68_], valine [v_71_] and tyrosine [v_72_] uptake rates are significantly higher for CSG+solASG condition as compared to CDG condition. Thus, we can correlate increased amino acid uptake rates and changes in glucose metabolism to hepatocellular function at the stable stage of culture.

### Metabolic Flux Analysis of Hepatocyte Cultures

Using a previously developed hepatic metabolic network comprising of 72 reactions and 27 extracellular metabolite measurements [45], [53], we performed a MFA for the different experimental conditions at 3 time points viz. the recovery stage, pre-stable stage and the stable stage of hepatocytes cultured in collagen single gel [CSG], cultured in collagen double gel sandwich configuration [CDG] and hepatocytes on collagen single gel with Adipogel overlay [CSG+solASG]. As shown in Figure 4, the estimated intracellular fluxes were compared by representation of differentially regulated fluxes for the CSG+solASG condition vs. CDG condition. MFA revealed a rich intermediary metabolic data for the different conditions with an increase certain reactions in the TriCarboxylic Acid [TCA] cycle [v_17_–v_20_] for the recovery stage comparison [Figure 4A]. At the pre-stable stage [Figure 4B], there was an increase in PPP [v_2_–v_9_] and reduction in gluconeogenic pathway [v_11_–v_13_], glutamine metabolism [v_36_–v_38_] and acetyl-CoA synthesis [v_30_,v_43_] and clearance [v_27_,v_42_] with increased citrate synthesis rate [v_15_] for the CSG+solASG condition as compared to the CDG condition. At the stable stage, increased amino acid uptake rates, decreased acetyl-CoA synthesis [v_30_, v_31_] with increased TCA cycle fluxes [v_17_–v_20_] were observed between the two conditions. Overall, from a functional standpoint the urea synthesis fluxes increased [v_21_,v_22_] and there were numerous differences in various pathway fluxes between the two conditions including an increase in albumin secretion for the CSG+solASG vs. CDG condition on all days of analysis.

We also performed a comparison of the CSG+solASG condition at different time points of the differentiation process. A comparison of the recovery stage and pre-stable stage cultures revealed significant increase in PPP and decrease in TCA cycle fluxes for the CSG+solASG condition with no significant difference in albumin synthesis rate [Table 5]. A significant decrease in glucose uptake, lipid metabolism and increase in pyruvate synthesis rates, amino acid uptake rates and albumin synthesis with increase in majority of the transamination reactions were observed for the stable stage vs. the recovery stage of cultures.

## Discussion

Primary hepatocytes are cultured *in vitro* using various techniques viz. collagen double gel sandwich [23]–[25], [29], [52], environmental perturbations [8], [11], [14], [16], [27], [42], encapsulation via cellular aggregation in 3D configuration [6], [11], [54] and cell-cell interactions [5]–[7], [20], [49]. Extracellular matrices have been shown to be implicated in cell polarity and functional maintenance of hepatocytes [27], [29], [31].

Particularly, there is some literature evidence for the effects of ECM proteins on enhanced albumin synthesis of hepatocytes [55] for individual ECM proteins viz. collagen I, collagen IV or laminin in the presence of either HGF or in co-cultures with fibroblasts. Moreover, it is well documented that collagen double gel sandwich cultures of hepatocytes, Matrigel single gel cultures or collagen-Puramatrix double gel cultures result in enhanced albumin synthesis rates and cytochrome P450 activities of hepatocytes as compared to collagen single gel cultures. This implies that ECM proteins have a metabolic effect on hepatocytes in culture [54]. Mathematical programming tools have been used as an additional step to elucidate the effect of environmental perturbations on hepatic function [43]–[46], [51], [56]. However, there is little information on the quantitative elucidation of the effect of ECM culture configurations on hepatocellular biochemical pathways.

While hepatic function is enhanced by utilizing the above mentioned culture systems, the albumin synthetic functional capacity of the cells is not maximal. In this study, Adipogel, a natural cell secreted extracellular matrix, can be added in soluble media format to hepatocyte cultures [Figure 1] and has been shown to be advantageous atleast from an albumin synthetic capacity standpoint [Figure 2B]. Preliminary results have shown that hepatocytes can be cultured on a single collagen gel layer with a double gel of solubilized Adipogel matrix for 10 days with enhanced albumin secretion rate as compared to CDG cultures [Figure 2B]. The application of a mammalian cell derived BME viz. Adipogel to the hepatocyte cultures in conjunction with elucidation of cell metabolism via mathematical programming techniques will assist in systematic identification of optimal nutrient and Adipogel supplementation for maximum hepatic function. This approach will also be conducive for adaptation of the optimized culture system for Bioartificial Liver Devices and drug toxicity studies.

In this context, we have performed a metabolic analysis of the different culture configurations viz. the collagen single gel [CSG], collagen double gel [CDG], collagen-Adipogel sandwich [CSG+ASG] and the collagen single gel+soluble Adipogel [CSG+solASG]. The analysis shows that the key amino acids upregulated for the CSG+solASG conditions are serine [v_69_], glycine [v_59_], isoleucine [v_61_], lysine [v_62_], leucine [v_63_], methionine [v_64_], arginine [v_68_], valine [v_71_] and tyrosine [v_72_] uptake rates as compared to the CDG condition at the stable stage of culture.

There is also an increased albumin synthesis rate for the CSG+solASG condition as compared to the CDG condition on day 10 of culture. The albumin flux is negligible and the amino acid fluxes contribute very little to albumin synthesis rates from a stoichiometric balance as also previously described using a similar metabolic network model [45]. However, increase in amino acid uptake rates with enhanced albumin synthesis rate is in agreement with literature evidence for perfused livers and cultured rat hepatocytes [39], [57]–[60]. Also, supplementation of branched chain amino acids such as leucine and isoleucine has been shown to increase albumin synthesis [60] consistent with our findings. However, literature evidence claims that synthesis of albumin is dependent on amino acid mediated modulation of translational initiation, inhibition of proteolysis, and activation of signal transduction pathways involving p70 S6 kinase, a key intermediate in protein synthesis initiation [61]. Thus, if the increased amino acid availability is responsible for enhanced albumin synthesis rate on day 10 of culture for the CSG+solASG condition as compared to the CDG condition, it is most likely due to above mentioned factors.

On the other hand, at the recovery stage and pre-stable stage of analysis, soluble Adipogel does not reveal increases in amino acid uptake rate as compared to the CDG condition probably due to a delayed response to Adipogel supplementation 48 hours before analysis. However, the albumin synthesis rate is significantly higher for the stable stage as compared to the recovery stage of hepatocyte cultures in CSG+solASG condition. Thus, a quantitative estimate of intracellular metabolic pathways can identify key pathways implicated in Adipogel supplementation of hepatocyte cultures and its effect on cell function.

**Figure 3:**
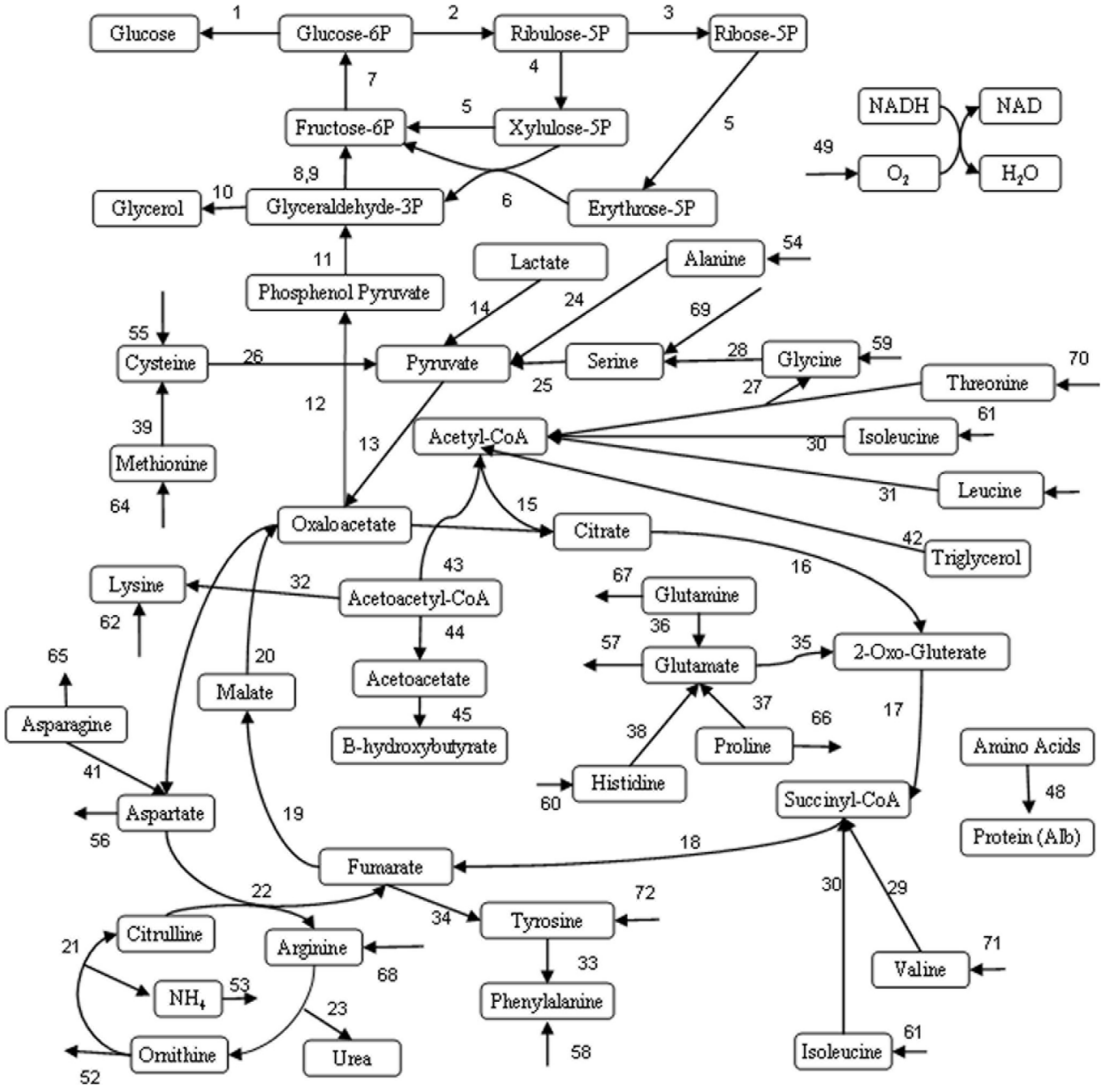
Metabolic Network Model for Hepatocyte Cultures. Arrows indicate direction of reaction assumed in the model. Numbers refer to reaction numbers listed in Table S1 and Table S2. Albumin reaction is not shown for purposes of clarity.

We have previously developed metabolic flux models of the perfused rat liver [41], [53] and hepatocytes [51], [62], including detailed stoichiometric balances of TCA and urea cycles, oxygen uptake, glycolytic/gluconeogeneic pathways, fatty acid metabolism, NADH and NADPH balance and albumin synthesis [Figure 3]. Based on the MFA model, we have investigated the metabolic fluxes of hepatocytes in the presence of Adipogel. The data obtained from the *in vitro* metabolite measurements has been utilized in a comprehensive metabolic network model to elucidate the effects of soluble Adipogel on hepatocyte intermediary metabolism and function. To obtain a temporal profile of the effects of extracellular matrix on cell metabolism, metabolite measurements were performed at the recovery stage, pre-stable stage and stable stage of primary hepatocyte cultures [Figure 1].

**Figure 4:**
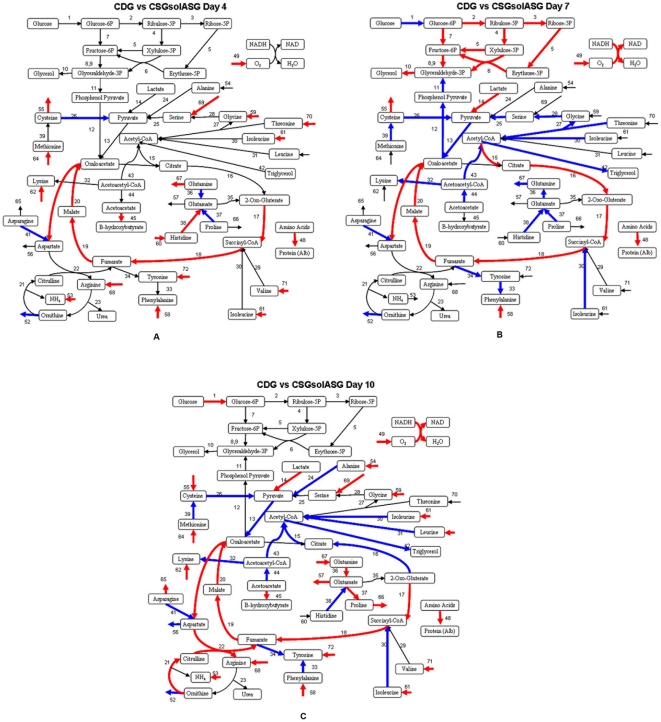
Metabolic Flux Analysis of Rat Hepatocytes *In vitro* Using Adipogel. [A] Recovery stage [B] Pre-stable stage and [C] Stable stage of culture. Hepatocytes cultured in five different configurations at a density of 500,000 cells/well in a 12 well plate; CSG corresponds to culture on single collagen gel; CDG corresponds to culture in collagen double gel sandwich configuration; CSG+solASG corresponds to hepatocytes cultured on collagen single gel with soluble Adipogel in the media; CSG+ASG corresponds to culture on collagen single gel with Adipogel overlaid on top;. Adipogel was utilized at a 1∶5 ratio with culture media and media was changed on days 0, 1,2,5,7 and 9. Metabolic Flux Analysis was performed on [A] recovery stage [B] pre-stable stage and [C] stable stage of culture. MFA results for CDG vs. CSG+solASG conditions are represented in the figures. *Note that the fluxes in red correspond to significantly upregulated fluxes [with statistical significance of p<0.05] for the CSG+solASG condition as compared to the CDG condition. Fluxes in blue correspond to significantly downregulated metabolite fluxes*.

For the CSG+solASG condition, differential effects on hepatic metabolism were observed on different days of analysis. As shown in Figure 4, different pathways were activated or downregulated during the culture period. While the albumin synthesis rate is significantly higher on all analysis days for the CSG+solASG condition vs. the CDG condition, the TCA cycle was significantly upregulated at the recovery stage [Figure 4A]; the PPP pathway was significantly upregulated at the pre-stable stage of culture [Figure 4B] while the amino acid uptake rates were significantly enhanced at the stable stage of culture as compared to the CDG condition [Figure 4C]. It is known from previous work that albumin and urea synthesis are competing pathways with gluconeogenesis for energy homeostasis in cultured hepatocytes [56]. Thus, the cells are undergoing alterations in metabolic phenotype over the culture period in comparison to the CDG cultures. At the recovery stage, the increase in TCA cycle fluxes might be compensating for reduced amino acid uptake that leads to enhanced albumin synthesis. At the stable stage, the increased amino acid uptake rate might contribute to enhanced albumin synthesis.

In general, the increase in transamination reaction fluxes with increased amino acid uptake rates might imply channeling of the amino acids towards albumin protein synthesis. Thus, the addition of these amino acids at early stages of the culture period in conjunction with Adipogel could potentially improve function significantly. Overall, enhanced albumin synthesis in the presence of Adipogel may be due to selective alterations in intracellular fluxes associated with these pathways that can be utilized for improving function.

### Conclusion and Future Work

Previous work referring to the development of a novel preadipocyte cell differentiation system has been utilized in this work for the generation of a natural basement membrane extract termed Adipogel with a unique extracellular matrix and growth factor composition [38].

We have shown that Adipogel can be utilized for augmenting hepatocyte differentiated function *in vitro* in combination with collagen over a 10 day period. In addition, the increase in amino acid metabolism in the presence of Adipogel shows promise in the integration of cellular metabolism with cell-extracellular matrix interactions. In order to elucidate the effects of Adipogel on intracellular hepatic function, we have utilized metabolite measurements for development of a Metabolic Flux Analysis model. These methodologies will provide a better understanding of the mechanistic implications of utilization of Adipogel for hepatocyte functional augmentation. This step will create the basis for an optimization based Flux Balance Analysis [FBA] model, such that the distribution of fluxes can be predicted with a limited number of values [active flux bounds] [51], [56]. The application of the FBA model to enhance hepatocellular function via. identification of media supplementation regimens will provide an additional step to improve hepatocellular functionality in Bioartificial Liver Devices.

## Supporting Information

Table S1 **List of reactions in metabolic network utilizing Gluconeogenic fluxes.** The common pathways include pentose phosphate pathway; lipid, glycerol and fatty acid metabolism; lactate metabolism and tricarboxylic acid (TCA) cycle; urea cycle; amino acid metabolism; oxygen uptake and electron transport and albumin protein metabolism.
(DOC)

Table S2 **List of reactions in metabolic network utilizing Glycolytic fluxes.** The common pathways include pentose phosphate pathway; lipid, glycerol and fatty acid metabolism; lactate metabolism and tricarboxylic acid (TCA) cycle; urea cycle; amino acid metabolism; oxygen uptake and electron transport and albumin protein metabolism.
(DOC)
