# Supplementary material for: Metabolic Profiling Based Quantitative Evaluation of Hepatocellular Metabolism in Presence of Adipocyte Derived Extracellular Matrix
Source: PLoS One. 2011 May 16;6(5):e20137. doi: 10.1371/journal.pone.0020137 (PMC3095641; doi:10.1371/journal.pone.0020137)
Supplement: Table S2 — List of reactions in metabolic network utilizing Glycolytic fluxes. The common pathways include pentose phosphate pathway; lipid, glycerol and fatty acid metabolism; lactate metabolism and tricarboxylic acid (TCA) cycle; urea cycle; amino acid metabolism; oxygen uptake and electron transport and albumin protein metabolism. (DOC) [file pone.0020137.s002.doc]

| Flux # | Reaction | Pathway |
| --- | --- | --- |
| **1** | **Glucose -> Glucose-6-phosphate** | **Glycolysis** |
| 2 | Glucose 6-phosphate ->2 NADPH + CO2 +Ribulose 5-P | PPP |
| 3 | Ribulose 5-P ↔ Ribose 5-P | PPP |
| 4 | Ribulose 5-P ↔ Xylulose 5-P | PPP |
| 5 | Ribose 5-P + Xylulose 5-P ↔ Fructose 6-P Erythrose 4-P | PPP |
| 6 | Erythrose 4-P + Xylulose 5-P ↔ Glyceraldehyde 3-P + Fructose 6-P | PPP |
| 7 | Glucose 6-phosphate ↔ Fructose 6-phosphate | Glycolysis |
| 8 | Fructose6-phosphate -> Fructose 1,6-Bisphosphate | Glycolysis |
| 9 | Fructose 1,6-Bisphosphate↔ 2 Glyceraldehyde 3-P | Glycolysis |
| 10 | Glycerol + ATP -> Glyceraldehyde 3-P +NADH | Lipid metabolism |
| 11 | Glyceraldehyde 3-P ↔ Phosphoenolpyruvate + ATP+NADH | Glycolysis |
| 12 | Phosphoenolpyruvate + ADP ↔ Pyruvate + ATP | Glycolysis |
| 13 | Pyruvate + CoA + NAD ↔ Acetyl-CoA + CO2 + NADH | Glycolysis |
| **14** | **Pyruvate + NADH ↔ Lactate** | **Lactate/TCA cycle** |
| 15 | Acetyl-CoA + Oxaloacetate -> Citrate | Lactate/TCA cycle |
| 16 | Citrate ↔ 2-oxo-Gluterate + NADH + CO2 | Lactate/TCA cycle |
| 17 | 2-oxo-Gluterate -> Succinyl-CoA + NADH + CO2 | Lactate/TCA cycle |
| 18 | Succinyl-CoA ↔ GTP + FADH2 +Furmarate | Lactate/TCA cycle |
| 19 | Furmarate ↔ Malate | Lactate/TCA cycle |
| 20 | Malate ↔ Oxaloacetate + NADH | Lactate/TCA cycle |
| 21 | Ornithine + CO2 + NH4 +2 ATP ->Citrulline | Urea cycle |
| 22 | Citrulline + Aspartate + ATP ->Arginine + Fumarate | Urea cycle |
| **23** | **Arginine -> Ornithine + Urea** | **Urea cycle** |
| 24 | Alanine + 2-oxo-Gluterate ↔ Pyruvate +Glutamate | AA metabolism |
| 25 | Serine -> Pyruvate + NH4 | AA metabolism |
| 26 | Cysteine + 2-oxo-Gluterate + HCN ↔Glutamate + HSCN + Pyruvate | AA metabolism |
| 27 | Threonine + ATP-> NADH + Glycine +Acetyl-CoA | AA metabolism |
| 28 | 2 Glycine ↔ Serine + CO2 + NH4 +NADH | AA metabolism |
| 29 | Valine + 2-oxo-Gluterate + ATP ->Glutamate + Succinyl-CoA + 2 NADH +FADH2 + CO2 | AA metabolism |
| 30 | Isoleucine + 2-oxo-Gluterate + ATP -> Glutamate + Succinyl-CoA + Acetyl-CoA + 2 NADH + FADH2 | AA metabolism |
| 31 | Leucine + 2-oxo-Gluterate -> Glutamate + NADH + FADH2 + ATP +Acetoacetate + Acetyl-CoA | AA metabolism |
| 32 | Lysine + 2 2-oxo-Gluterate -> 2 Glutamate + 3 NADH + FADH2 + 2CO2 + Acetoacetatyl-CoA | AA metabolism |
| 33 | Phenylalanine + O2 -> Tyrosine | AA metabolism |
| 34 | Tyrosine + 2-oxo-Gluterate + 2 O2 -> Glutamate + CO2 + Fumarate + Acetoacetate | AA metabolism |
| 35 | Glutamate ↔ 2-oxo-Gluterate + NADPH +NH4 | AA metabolism |
| 36 | Glutamine -> Glutamate + NH4 | AA metabolism |
| 37 | Proline -> Glutamate + NADH | AA metabolism |
| 38 | Histidine -> NH4 +Glutamate | AA metabolism |
| 39 | Methionine +3 ATP + Serine -> Cysteine + NADH + Succinyl-CoA + NH4 | AA metabolism |
| 40 | Oxaloacetate + NH4 +NADH ↔Aspartate | AA metabolism |
| 41 | Asparagine ↔ Aspartate + NH4 | AA metabolism |
| 42 | Triacylglycerol + 4 ATP -> Glyceraldehyde 3-P + 24 Acetyl-CoA +21 FADH2 + 22 NADH | Lipid metabolism |
| 43 | 2 Acetyl-CoA ↔ Acetoacetyl-CoA | Lipid metabolism |
| 44 | Acetoacetyl-CoA -> Acetoacetate | Lipid metabolism |
| **45** | **Acetoacetate + NADH ↔Hydroxybutyrate** | **Lipid metabolism** |
| 46 | NADH + 0.5 O2 -> NAD | Electron transport |
| 47 | FADH2 + 0.5 O2 -> FAD | Electron transport |
| **48** | **Albumin Synthesis** | **Protein metabolism** |
| **49** | **O2 Input** | **Electron transport** |
| **50** | **CO2 Output** | **Electron transport** |
| **51** | **Acetoacetate Output** | **Lipid metabolism** |
| **52** | **Ornithine Output** | **Urea cycle** |
| **53** | **Ammonia Output** | **Urea cycle** |
| **54** | **Alanine Output** | AA metabolism |
| **55** | **Cysteine Output** | AA metabolism |
| **56** | **Aspartate Output** | AA metabolism |
| **57** | **Glutamate Output** | AA metabolism |
| **58** | **Phenylalanine Output** | AA metabolism |
| **59** | **Glycine Output** | AA metabolism |
| **60** | **Histidine Output** | AA metabolism |
| **61** | **Isoleucine Output** | AA metabolism |
| **62** | **Lysine Output** | AA metabolism |
| **63** | **Leucine Output** | AA metabolism |
| **64** | **Methionine Output** | AA metabolism |
| **65** | **Asparagine Output** | AA metabolism |
| **66** | **Proline Output** | AA metabolism |
| **67** | **Glutamine Output** | AA metabolism |
| **68** | **Arginine Output** | **Urea cycle** |
| **69** | **Serine Output** | AA metabolism |
| **70** | **Threonine Output** | AA metabolism |
| **71** | **Valine Output** | AA metabolism |
| **72** | **Tyrosine Output** | AA metabolism |
